# Donor Site Morbidity in Unilateral and Bilateral Transverse Musculocutaneous Gracilis (TMG) Flap Breast Reconstruction: Sensation, Function, Aesthesis and Patient-Reported Outcomes

**DOI:** 10.3390/jcm10215066

**Published:** 2021-10-29

**Authors:** Laura Cosima Siegwart, Anca Bolbos, Valentin Felix Haug, Yannick Fabian Diehm, Ulrich Kneser, Dimitra Kotsougiani-Fischer

**Affiliations:** Department of Hand, Plastic and Reconstructive Surgery, Microsurgery, Burn Center, BG Trauma Center Ludwigshafen, Hand and Plastic Surgery, University of Heidelberg, 67071 Ludwigshafen, Germany; lauracosima.siegwart@bgu-ludwigshafen.de (L.C.S.); anca.bolbos@yahoo.de (A.B.); valen-tin.haug@bgu-ludwigshafen.de (V.F.H.); yannick.diehm@gmx.de (Y.F.D.); ulrich.kneser@bgu-ludwigshafen.de (U.K.)

**Keywords:** TMG flap, transverse musculocutaneous gracilis flap, donor site morbidity, breast reconstruction, autologous breast reconstruction, breast cancer

## Abstract

The transverse musculocutaneous gracilis (TMG) flap has become a popular choice for breast reconstruction. This study aimed to compare the donor site morbidity in unilateral and bilateral procedures. Patients receiving a TMG flap (January 2008–October 2019) were invited to a follow-up and grouped according to unilateral (UL group) or bilateral (BL group) breast reconstruction. Outcome criteria included sensation, function and aesthesis of the thighs. Patient-reported outcomes were surveyed using validated questionnaires. The number and kind of refinement procedures for aesthetic purposes on the donor thighs were evaluated. Thirty-eight patients with 59 TMG flaps were included in the study (UL group: *n* = 17, BL group: *n* = 21). Normal to slightly diminished superficial skin sensation was maintained in most of the thigh skin (98.4%). Strength and mobility were without impairment in >80% of the thighs in both groups. Thigh symmetry was achieved in both groups. Symmetrisation procedures were significantly more often performed in the UL group (*p* = 0.005). The total number of refinement procedures was similar in both groups. Patient-reported outcomes were similar with good appearance of the thighs and scars, excellent function and low pain levels. The TMG flap offers excellent function and sensation on the donor thigh. Thigh symmetry and good patient satisfaction may be achieved in both unilateral and bilateral breast reconstructions.

## 1. Introduction

Breast reconstruction following mastectomy has become an integral component of comprehensive breast cancer care. Due to consistent improvements, free flap breast reconstruction has evolved to be a popular choice, creating natural breasts with no risk for implant-associated complications and superior quality of life compared to silicone implant breast reconstruction [1]. Abdominal-based free flaps, such as the deep inferior epigastric perforator (DIEP) flap or the muscle-sparing transverse rectus abdominis (MS-TRAM) flap represent the gold standard [2,3]. However, when the lower abdomen does not provide enough tissue excess, the thighs and the buttocks should be considered as alternative donor sites [4,5]. The transverse musculocutaneous gracilis (TMG) flap from the thigh has been utilized for both unilateral and bilateral breast reconstruction. The reliable anatomy with no need for preoperative imaging, the ease of flap raising, the quality and plasticity of the soft tissue as well as the excellent outcome of the breasts with appealing shape and body-appropriate volume represent various advantages of the TMG flap [6,7,8]. However, limited evidence exists regarding sensation, function and aesthesis of the donor thigh in TMG flap breast reconstruction. Some studies have reported skin sensation loss and impaired thigh function, genital changes and asymmetry of the thigh contour [9,10]. Unfortunately, the inhomogeneity of non-objective outcome measurements used in previous studies impedes reliable conclusions on TMG donor site morbidity [11]. This study aimed to evaluate and compare donor site morbidity including sensation, function, aesthesis and patient-reported outcomes in unilateral and bilateral TMG flap breast reconstruction.

## 2. Materials and Methods 

Following approval of the local Ethical Committee (2018-13902_1), a retrospective study was designed in accordance with the ethical standards laid down in the World Medical Association Declaration of Helsinki (June 1964) and its later amendments.

All patients who received unilateral or bilateral TMG flap breast reconstructions following successful therapeutic or prophylactic mastectomy from 1 January 2008 to 31 October 2019 in our academic hospital were identified. Patients were contacted by telephone or mail and invited to participate in a prospective clinical follow-up. Eligible patients who gave written informed consent were included in the study and grouped according to unilateral (UL group) or bilateral (BL group) breast reconstruction. Patients with previous surgery on the lower extremity were excluded from study participation. TMG flap breast reconstructions were performed according to a standardized in-house protocol, applying a two-team approach of simultaneous flap harvest and recipient site preparation. The optimized surgical technique for TMG flap harvest has been published previously [6]. All bilateral breast reconstructions were performed in two separate surgeries with a distance of at least 2 months between the procedures. The primary outcomes were donor site function, sensation and aesthetics including scar position and thigh symmetry, as well as patient-reported outcomes questioning satisfaction with the inner thigh and scarring, lower extremity function, sexual function and pain. Secondary outcomes were the number and kind of refinement procedures for aesthetic purposes on the donor thighs. The postoperative follow-up constituted a minimum of 3 months after TMG flap breast reconstruction.

Patient charts and the electronic inpatient hospital information system were screened for data acquisition. Demographic patient data (age, body mass index (BMI)), comorbidities (breast cancer, genetic predisposition to breast cancer), risk factors (smoking status, diabetes mellitus, preoperative chemotherapy or radiotherapy), indication for reconstruction of the breast (therapeutic or prophylactic mastectomy, breast aplasia), intraoperative data (unilateral or bilateral procedure, operation time) and the number and kind of secondary refinement operations for aesthetic purposes on the donor thigh were extracted. 

**Figure 1:**
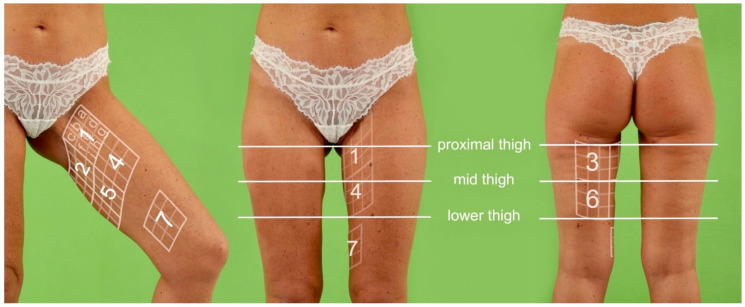
Normal weight female patient (47 years, BMI 22.7 kg/m^2^) with bilateral transverse musculocutaneous gracilis (TMG) flap breast reconstruction. Digital markings indicating the grid for sensation measurements on the donor thigh and the proximal thigh, the mid-thigh and the lower thigh, where measurement of the thigh circumference was conducted. The grid for sensation measurements was divided in euals quadrants (1–7). Each quadrant was subdivided into nine smaller squares (a–i).

To evaluate skin sensation, the donor thigh was divided into seven equal quadrants (quadrants 1–7), creating a grid for sensation measurements. First, a line was drawn extending the scar by 2 cm on its anterior and posterior aspect. Second, the length of this line was divided by three, defining the length and height of each quadrant. Third, a total of six quadrants were marked on the patient’s skin (quadrants 1–6). In addition, a quadrant was marked on the medial thigh proximal to the patella (quadrant 7), representing the sensory area of the obturator nerve. Then, each quadrant was subdivided into nine smaller squares (a–i). The grid for sensation measurement on the donor thigh is shown in Figure 1. 

For each square, the pressure thresholds of slowly adapting fibers with Semmens Weinstein monofilaments were examined, as previously shown by Visconti et al. on the lower abdomen donor site in DIEP flap breast reconstruction [12]. A standard kit composed of five monofilaments (Baseline Tactile monofilament evaluators, Item: 12-1638, Fabrication Enterprises, White Plains, NY, USA) with increasing target force was used, as usually applied for testing pressure thresholds in the hands. Target forces of the Semmens Weinstein monofilaments were assigned to corresponding qualities of skin sensitivity. Examination of motoric thigh function included measurement of adduction strength and hip joint adduction and abduction mobility of both legs. The adduction strength was examined using the Medical Research Council’s scale for muscle strength ranging from M0 (no muscle movement) to M5 (full muscle strength against full resistance) [13]. Goniometric measurement using the neutral zero method was applied to examine hip joint adduction and abduction. The thigh circumference was measured on proximal thigh, mid-thigh and lower thigh to evaluate thigh symmetry, as shown in Figure 1. In the UL group, the circumference of the donor leg was set in relation to the circumference of the non-operated leg. In the BL group, the circumference of the smaller leg was set in comparison to the larger leg. In addition, the scar length and scar position to the groin were measured (quadrant 2, square b).

Validated questionnaires, including the Lower Extremity Function Scale and the BODY-Q were used to measure patient-reported outcomes. The Lower Extremity Function Scale is a validated measurement tool for assessing difficulties in daily activities that require lower extremity function, such as walking or using the stairs [14]. The maximum score (80 points) indicates a good leg function, while the minimum score (0 points) indicates strong disability of the leg function. The BODY-Q is composed of validated scales measuring patient-reported outcomes related to body contouring procedures [15]. Independent appearance scales were used to survey satisfaction with the donor thigh (BODY-Q Satisfaction with inner thighs) and donor site scar (BODY-Q Appraisal of scars). BODY-Q Satisfaction with the inner thighs is a 4-item scale. Items ask how smooth and toned the inner thighs are, how the skin looks and how the inner thighs look when naked. BODY-Q Appraisal of scars is a 10-item scale. Items ask about being bothered by the width, location, length and color of the scars, as well as how noticeable the scars are. The BODY-Q sexual well-being is a health-related quality of life scale, which is composed of 5 items. Items ask about satisfaction with sex life, being comfortable with the lights on during sex, feeling sexually attractive when undressed, etc. On each BODY-Q scale, scores range from 0 to 100 and high scores reflect high patient satisfaction. The numerical rating scale (NRS) (0–10) was used to survey pain on the donor thigh at rest and during movement. Zero represented “no pain” and 10 represented the “maximum pain”. In addition, patients were asked about changes in the genital area, such as labial spreading after TMG flap breast reconstruction with dichotomous answer options (yes/no) and whether they would undergo TMG flap breast reconstruction again (yes/no).

Data are presented as frequencies for categorical variables, means and SD for normally distributed variables and median and range for not normally distributed variables. Outcomes were compared using the Fisher exact test (*n* < 5 per crosstable) or the chi-square test for categorical variables (*n* > 5 per crosstable), the two-sided unpaired T-test for normally distributed continuous variables and the Mann–Whitney U test for not normally distributed variables. All data analyses were performed using Prism 8.3.0 software (GraphPad Software, San Diego, CA, USA). Statistical significance was set at *p* < 0.05.

## 3. Results 

In total, 52 Caucasian patients received 82 TMG flaps for breast reconstruction. Thirty-eight eligible patients with 59 TMG flap breast reconstructions were included in the study with an inclusion rate of 73% (38/52). Reasons for study exclusion were death (*n* = 2), refusal to participate in the study (*n* = 4) and missing or incorrect contact information (*n* = 8). Seventeen patients (45%) received unilateral and 21 patients (55%) bilateral TMG flap breast reconstructions. The follow-up time was median 34 months in the UL group and 54 months in the BL group. The analysis of patient characteristics showed an equal distribution with regard to age, BMI, comorbidities and risk factors between groups. In the BL group, genetic predisposition to breast cancer and the rate of prophylactic mastectomies were significantly increased (*p* < 0.001 and *p* < 0.001). Patients in the UL group received significantly more TMG flap breast reconstruction due to breast cancer and therapeutic mastectomy (*p* = 0.002). The total flap success rate was 94.9%. Comparison of patient characteristics is shown in Table 1.

**Figure 2:**
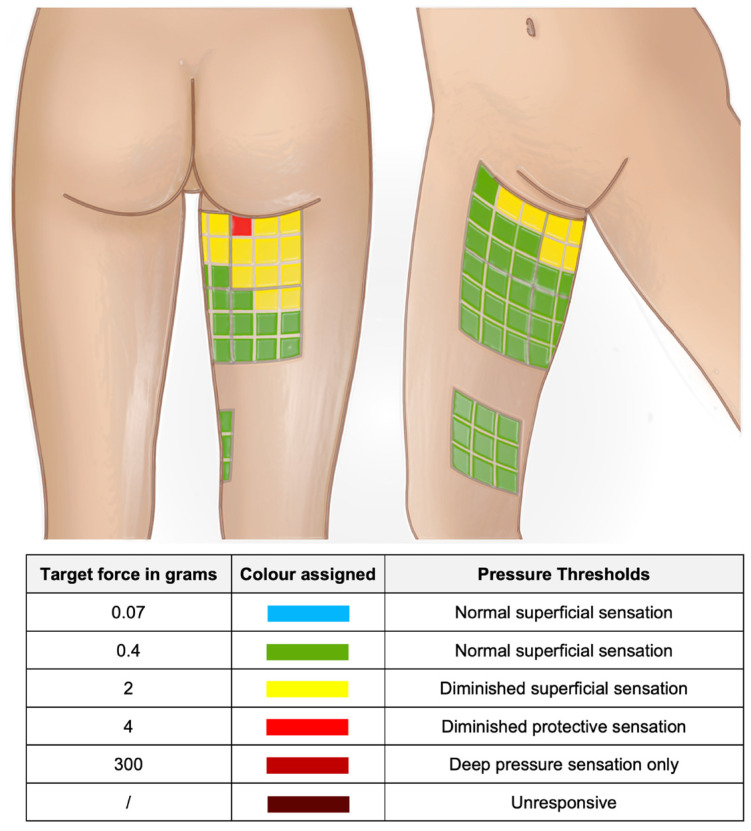
Skin sensation on the donor thigh. For each of the seven quadrants on the donor thigh, the average pressure thresholds have been examined and were categorized with colors, as indicated in the legend. According to the average pressure threshold, each square in the quadrant has been colored.

In the follow-up examination, most of the donor thigh skin (44/63 squares, 69.8%) showed normal sensation (median target force 0.4 g) including quadrants 4 and 5, which are supplied by the anterior femoral cutaneous nerve, and quadrant 7, which is supplied by the obturator nerve. Diminished superficial sensation (median target force 2 g) was measured in quadrant 1, 2, 3 and 6 on the proximal thigh and dorsal thigh (18/63 squares, 28.6%). Diminished protective sensation (median target force 4 g) was measured close to the scar in quadrant 3 (1/63 squares, 1.6%), in the sensory area of the posterior femoral cutaneous nerve. No deep pressure sensation or complete loss of sensation was found. Results of skin sensation examination are shown in Figure 2.

Function, including hip adduction strength and hip mobility, was similar in both groups. Ninety-three percent (55/59) of the examined donor legs showed full hip adduction strength (Medical Research Council’s grade M5). In 7% (4/59) of the donor legs, hip adduction strength was slightly reduced with inability to perform active hip adduction against full resistance (Medical Research Council’s grade M4). The median hip adduction was 25° and the median hip abduction was 30° in goniometric measurement in both groups. A reduced hip adduction ≥10° of the donor leg compared to the contralateral leg was measured in in 5.9% (1/17) and 4.8% (1/21) of the patients in the UL and BL group without a statistical difference (*p* = 0.999). Moreover, a reduced hip abduction ≥10° of the donor leg compared to the contralateral leg was measured in 11.8% (2/17) of the patients in the UL group and in 9.5% (2/21) in the BL group with again no statistical difference (*p* = 0.999). The results of the function are shown in Table 2. 

Aesthetic refinement procedures were performed in 41.1% of the donor thighs in the UL group and in 23.8% of the donor thighs in the BL group, with no statistical difference. In the UL group, contralateral thigh alignment by liposuction or thigh lift (23.5%) for symmetrisation and contour alignment of the donor thigh (11.8%) were the most common refinement procedures. Symmetrisation procedures of the thighs were significantly more often performed in the UL group compared to the BL group (*p* = 0.005). In the BL group, scar correction (11.9%) and dog ear excision (11.9%) represented the most common refinement procedures of the donor thighs. All aesthetic refinement procedures of the thighs were combined with breast touch-up procedures in both groups. Refinement procedures of the donor site are summarized in Table 3.

**Figure 3:**
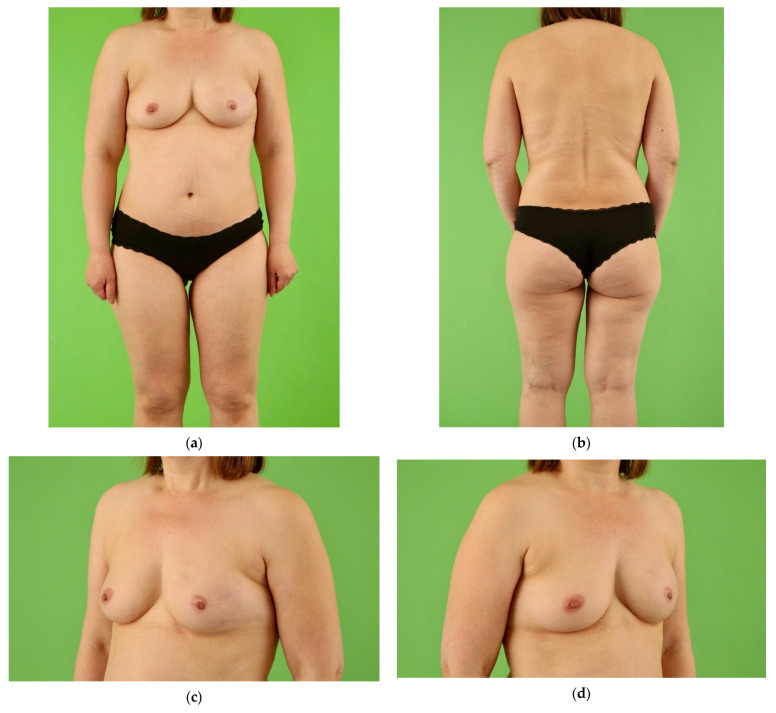
Normal-weight female patient (49 years, BMI 24.9 kg/m^2^) with invasive ductal carcinoma on the left breast in the medical history. Salvage reconstruction of the left breast with unilateral TMG flap from the right thigh following implant failure and skin-sparing mastectomy of the left breast. One refinement procedure of lipofilling from the abdomen was performed for volume boost-up the left breast combined with excision of the TMG skin island. In a second procedure, unilateral nipple reconstruction was conducted with nipple-sharing. Postoperative view at 6.0 year follow-up. (**a**) Front view with natural symmetry of the thighs after unilateral TMG flap harvest from the right thigh and concealed donor site scar in the groin of the right thigh. Excellent symmetry of both breasts following TMG flap reconstruction of the left breast and natural right breast; (**b**) Back view with natural symmetry of the thighs and concealed donor site scar in the natural crease of the right buttock; (**c**,**d**) Natural shape with volume symmetry of both moderate size breasts following unilateral TMG flap reconstruction of the left breast.

**Figure 4:**
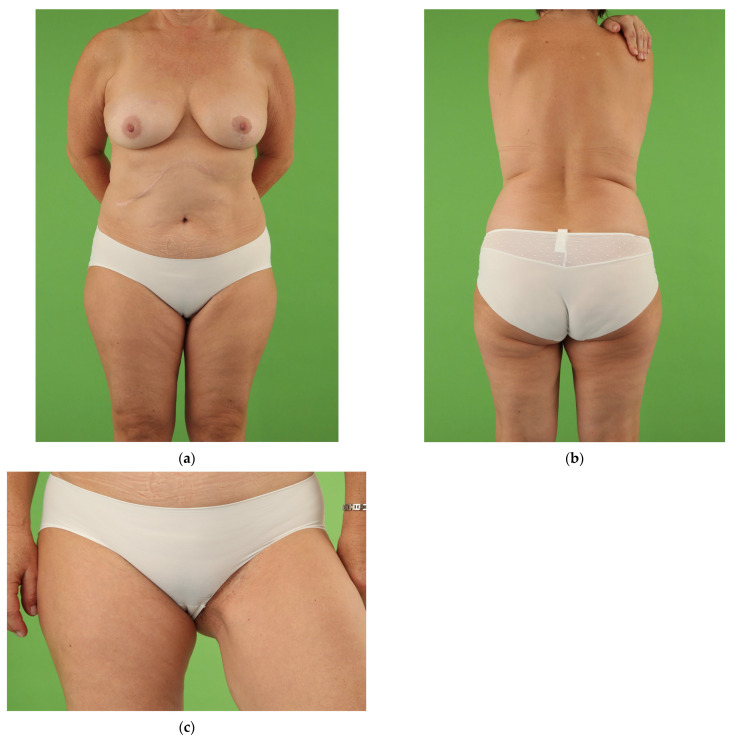
Overweight female patient (59 years, BMI 26.4 kg/m^2^) with invasive ductal carcinoma of the right breast and partial hepatectomy in the medical history. Salvage reconstruction of the right breast with TMG flap from the left thigh following implant failure after skin-sparing mastectomy and immediate silicone implant reconstruction. Breast reduction with vertical scar of the left breast for symmetrisation. Postoperative view at 5.0 year follow-up. (**a**) Front view with excellent shape and symmetry of both medium size breasts following TMG flap breast reconstruction of the right breast. Natural symmetry of the thighs after unilateral TMG flap harvest with secret scar in the groin of the left donor thigh; (**b**) Back view with secret scar in the natural crease of the left donor thigh; (**c**) Appealing contour of the left donor thigh with concealed scar in the groin.

**Figure 5:**
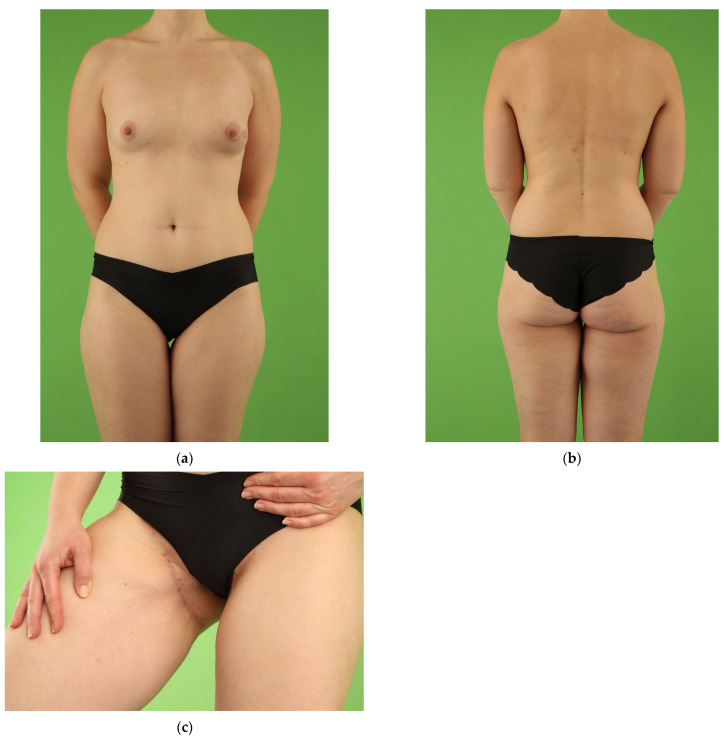
Normal weight female patient (39 years, BMI 22.9 kg/m^2^) with positive BRCA mutation status. Nipple-sparing mastectomy of the left breast and immediate breast reconstruction with TMG flap from the right thigh. Nipple-sparing mastectomy of the right breast and immediate breast reconstruction with TMG flap from the left thigh is scheduled. Postoperative view at 0.5 year follow up. (**a**) Front view with appealing shape and symmetry of both small size breasts following TMG flap breast reconstruction of the left breast. TMG skin island visible on the outer left breast. Natural symmetry of the thighs after unilateral TMG flap harvest with secret scar in the groin of the right donor thigh; (**b**) Back view with secret scar in the natural crease of the right donor thigh; (**c**) Excellent shape of the right donor thigh with active scar in concealed position in the groin.

At the follow-up examination the scar length and position in or slightly beneath the groin was similar in both groups. The UL group showed persistent smaller mean circumferences of the donor thigh compared to the non-operated thigh on the level of the proximal thigh, mid-thigh and lower thigh. However, there was no statistical difference. Outcomes of the donor thigh and breast in unilateral TMG flap breast reconstruction are shown in Figure 3, Figure 4 and Figure 5. 

In the BL group, the thigh circumference of both legs was almost equal on the proximal thigh, mid-thigh and lower thigh with again no significant differences. The results of the measurements are shown in Table 4. 

The Lower Extremity Function Scale revealed an excellent function of the lower extremity with almost maximum function in both groups (UL group 77 points vs. BL group 78.5 points; *p* = 0.120). The BODY-Q appearance scales showed good scores for the satisfaction with the thighs and appraisal of the scars, which were similar for both groups (BODY-Q Satisfaction with inner thighs: UL group 66 points vs. BL group 66 points, *p* = 0.487; BODY-Q Appraisal of scars: UL group 65 points vs. BL group 66 points; *p* = 0.791). In both groups, the BODY-Q showed diminished sexual function, which was reflected by an intermediate score (UL group 47 points vs. BL group 49 points, *p* = 0.902). Most of the patients (92.1%) expressed having no pain and a few patients (7.8%) reported having minor pain (NRS 0–4) on the donor thigh at rest. In addition, a few patients (7.8%) stated to have pain when sitting. During movement, 63.2% of the patients stated that they have no pain on the donor thigh and 26.3% of the patients stated that they have minor pain (NRS 0–4). Ten percent (10.5%) of the patients expressed that they have moderate pain during movement (NRS 5–8). Five percent (5.2%) of the patients reported taking pain medication due to donor site pain. There was no significant difference between the UL and BL group. Eight percent (7.9%) of the patients reported the appearance of labial spreading since TMG flap breast reconstruction. There was no significant difference in comparison of the UL and BL group (*p* = 0.577). All patient-reported outcome measures are summarized in Table 5.

In both groups, >85% of the patients stated that they would undergo TMG flap breast reconstruction again (unilateral group 94.1% (16/17) vs. bilateral group 85.7% (18/21), *p* = 0.613). 

## 4. Discussion

The TMG flap has become a popular choice for unilateral and bilateral free flap breast reconstruction in patients with slim and regular body types with a success rate of up to 99% [11]. This study found a low long-term donor site morbidity as it relates to function and sensation, aesthetic appearance and patient-reported outcomes. Hip joint strength and mobility were without impairment in >80% of the thighs and 98.4% of the thigh skin maintained normal to slightly diminished skin sensation. Moreover, excellent thigh symmetry was achieved in unilateral and bilateral TMG flap breast reconstructions. However, in unilateral TMG flap breast reconstructions, aesthetic refinement procedures of the contralateral thigh were required to achieve thigh symmetry. Patient perceptions regarding appearance of the thigh and donor site scars were good. Lower extremity function was reported to be excellent and donor site pain was a minor concern. 

The safety and applicability of the TMG flap have been outlined in plenty of studies [5,8,16,17]. The TMG flap is based on the dominant pedicle to the gracilis muscle and its perforators nourishing the excess of fat and skin laxity harvested from the medial thigh [18]. Preoperative Doppler probe examination eases the precise localization of the dominant pedicle and may shorten the flap harvest and operation time. Recently, Schwaiger et al. reported on the safe use of the TMG flap for breast reconstruction in patient populations with BMI > 25 kg/m^2^ expanding its applications for breast reconstruction [17]. Moreover, Weitgasser et al. conducted the first head-to-head comparison of double DIEP flaps and double TMG flaps for simultaneous breast reconstruction, emphasizing the rising value of the TMG flap for bilateral breast reconstruction [19]. However, there is a clear lack of evidence on long-term sensation, function and aesthetic appearance of the donor site in TMG flap breast reconstruction. In the past, various studies reported on disturbed sensation of the donor thigh in 0% to 74% of the patients with TMG flaps for breast reconstruction [10,16,20]. Reduced or abnormal sensation was located on the dorsal thigh [7,21], the medial thigh or around the donor site scar [10,16,20]. However, previous studies have clear limitations since subjective tools were applied to assess sensory function or the means of the investigation were not explained. 

The authors are conscious that sensation is a complex sense that includes touch, pain, temperature, vibration, and proprioception. In this study, we used pressure sensitivity measurement applying Semmes–Weinstein monofilaments which present a standardized objective tool to examine sensory function in free flap breast reconstruction [12,22]. We confirmed that most of the skin on the anterior and lower thigh maintained normal sensation. The reduced sensation was limited to the skin on the proximal thigh close to the scar and the dorsal thigh in the sensory area of the posterior cutaneous femoral nerve. In the future, refinements in flap raising techniques might succeed in retaining normal sensation on the posterior thigh by protecting the posterior femoral cutaneous nerve and its skin branches. Similarly, surgical refinements reduced the diminished skin sensation around the umbilicus and scar on the lower abdomen in DIEP flap breast reconstruction [12]. We also surveyed pain on the donor thigh using the NRS (0–10) as a validated instrument. Fortunately, more than 90% of the patients reported having no pain at rest and only a few patients reported having pain when sitting, which is a major complaint of free flaps from the buttocks, such as the inferior gluteal artery perforator (IGAP) flap [23] In addition, more than 60% of the patients stated that they have no pain in movement and no patient had regular need for pain medication.

In 1994, Deutinger et al. showed a slight loss in hip adduction strength of 11% after gracilis muscle elevation for other reconstructive purposes in a dynamometric measurement [24]. To examine whether this quantitative loss in function translates into lifestyle limitations, we performed a follow-up examination. In our study, 93.2% of the patients accomplished full hip adduction strength, and hip joint adduction and abduction mobility was within age-adjusted limits, irrespective of flap laterality. In addition, both patient cohorts scored >95% of maximum function in the Lower Extremity Function Scale, which compares to normative data [25]. Similarly, Fricke et al. stated no loss of function in patients’ everyday life following gracilis muscle harvest for free flap microsurgery [26]. The excellent function of the lower extremity in both unilateral and bilateral TMG flap breast reconstruction might be explained by the accessory role of the gracilis muscle, in particular in comparison to the thigh adductor muscles. This aspect is serious because return to full function and complete physical recovery is a key component of quality of life. Therefore, recovery should be optimized in breast cancer survivors through early physical therapy, which has been shown to be an advantageous element in post-reconstruction breast cancer care [27,28]. 

In this study, we were able to show that thigh symmetry was achieved in almost all patients in unilateral and bilateral TMG flap breast reconstruction. However, patients who received unilateral TMG flap breast reconstruction likely needed secondary refinement procedures for symmetrisation of the contralateral thigh. Our results confirm the concealed scar position on the donor thigh with a median distance of 1 cm to the groin. Similarly, a pleasing secret scar may be achieved using a single horizontal thigh-lift-type approach for free gracilis muscle harvest for other reconstructive purposes [29]. However, due to migration or inferior scar quality scar correction was the second most common refinement procedure on the donor thigh. All refinement surgeries on the donor thigh were combined with touch-up procedures to optimize the breast outcome. Notably, in DIEP flap breast reconstruction, aesthetic refinement procedures are performed in up to 45% of all breast reconstructions to enhance the lower abdominal donor site [30,31]. 

Unfortunately, the BREAST-Q, which is a validated instrument to survey patients’ perception in breast reconstruction, was unsuitable to examine patient satisfaction in our study. This is because the BREAST-Q reconstructive module are tailored to abdominal-based or implant-based breast reconstructions and do not consider alternative donor sites, such as the medial thigh [32]. Therefore, we utilized the validated BODY-Q to evaluate patient satisfaction with the aesthetic outcome of the donor thigh and the scar. The BODY-Q Satisfaction with inner thighs and BODY-Q Appraisal of scars showed good satisfaction with the appearance of the medial thigh and donor site scar with no difference between the both groups. In contrast to the POSAS score, the BODY-Q Appraisal of scars evaluates multiple dimensions of the scar such as scar location, length, how noticeable the scars are as well as scar quality. Therefore, the BODY-Q Appraisal of scars is particularly valuable for future comparison with other donor sites, e.g., the PAP flap donor site. However, Opsomer et al. recently adapted the BREAST-Q without any psychometric evaluation to compare the patient-reported donor site outcome between the superior gluteal artery perforator (SGAP) flap, lumbar artery perforator (LAP) flap and DIEP flap [33]. 

To date, few studies pointed to genital changes in TMG flap breast reconstruction [10,16]. We surveyed this omitted topic in both patient groups with a non-validated question. A few patients (7.9%) reported having labial spreading since TMG flap breast reconstruction, which might be due to extensive soft tissue harvest on the inner thighs or contracture of the donor site scar. Similarly, Pülzl et al. reported “a little” change in the genital region in 11% of patients, and Craggs et al. surveyed genital changes in 24% of the patients [10,16]. In this context, the BODY-Q questionnaires showed an intermediate score of sexual well-being in both patient cohorts. However, diminished sexual function represents an elusive finding in breast cancer survivors which might be due to the primary disease, lack of satisfaction with breasts, or the donor site or due to other concerns. Similarly, in a prospective study, Razzano et al. showed that sexual well-being was the lowest reported outcome one year after reconstruction in patients with unilateral DIEP flap breast reconstructions when using the validated BREAST-Q score [34]. 

Even though this study advances the literature about donor site morbidity and patient-reported outcomes in unilateral and bilateral TMG flap breast reconstruction, limitations of the study should be considered. Due to its retrospective design, data extraction and clinical follow-up were limited to patients willing to participate in the study. This may have biased our findings. In the future, prospective studies should be conducted to confirm the results of our study. Moreover, future prospective studies should objectively compare the TMG flap to the profundal artery perforator (PAP) flap, which might provide several advantages to the TMG flap regarding both the reconstructed breast and the donor site. 

## 5. Conclusions

The TMG flap offers a low long-term donor site morbidity with excellent motoric and sensory outcomes. Thigh symmetry may be achieved in both unilateral and bilateral TMG flap breast reconstructions. However, patients should be educated about the likelihood of refinement procedures to optimize thigh symmetry in unilateral TMG flap breast reconstruction. Objective evaluation of patient-reported outcomes showed good patient satisfaction with the appearance of the thighs and scars and full thigh function with minimal pain. Our findings should assist surgeons and patients alike in counseling and decision-making on the best individual option in free flap breast reconstruction including the TMG flap as an excellent option for unilateral and bilateral breast reconstruction in selected patients. 

## Figures and Tables

**Table 1:** Patient characteristics.

	UL Group	BL Group	*p*-Value
Patients, N (%)	17 (44.7%)	21 (55.3%)	NA
TMG flaps, N (%)	17 (28.8%)	42 (71.2%)	NA
Age (years), Mean (SD)	50.6 (11.7)	48.6 (10.4)	0.566
BMI (kg/m^2^), Median (R)	23.5 (20.7–32.8)	24.7 (19.2–32.3)	0.994
Breast cancer, N (% of patients)	14 (82.4%)	16 (76.2%)	0.708
Genetic predisposition to breast cancer, N (% of patients)			
Total	4 (23.5%)	18 (85.7%)	<0.001 *
Breast cancer	2 (11.7%)	12 (57.1%)	NA
No breast cancer	2 (11.7%)	6 (28.6%)	NA
Risk Factors, N (% of patients)			
Diabetes Mellitus	0 (0%)	1 (4.8%)	0.999
Obesity (BMI ≥ 30 kg/m^2^)	2 (11.8%)	4 (19.0%)	0.672
Active smoker	3 (17.6%)	4 (19.0%)	0.999
Preoperative chemotherapy	9 (52.9%)	11 (52.4%)	0.744
Preoperative radiotherapy	8 (47.1%)	11 (52.4%)	0.744
Indication for reconstruction, N (% of breasts)			
Therapeutic mastectomy	14 (82.4%)	16 (38.1%)	0.002 *
Prophylactic mastectomy	2 (11.8%)	26 (61.9%)	<0.001 *
Breast aplasia	1 (5.9%)	0 (0%)	0.288
Follow-up (months), Median ®	34 (4–117)	54 (24–144)	0.081

NA, not applicable; UL, unilateral; BL, bilateral; SD, standard deviation; BMI, body mass index; TMG, transverse musculocutaneous gracilis; R, range; N, number; * indicates significant difference (*p* < 0.05).

**Table 2:** Lower extremity function.

	UL Group	BL Group	*p*-Value
Patients, N (%)	17 (44.7%)	21 (55.3%)	NA
Donor sites, N (%)	17 (28.8%)	42 (71.2%)	NA
Hip adduction strength, N (% donor legs)			
MRC 1–3	0 (0%)	0 (0%)	0.999
MRC 4	3 (17.6%)	1 (2.4%)	0.068
MRC 5	14 (82.4%)	41 (97.6%)	0.068
Hip mobility (degree), Median (R)			
Adduction	25 (15–35)	25 (15–40)	0.903
Abduction	30 (20–45)	30 (20–45)	0.435
Impairment of hip mobility, N (% of patients)			
Adduction	1 (5.9%)	1 (4.8%)	0.999
Abduction	2 (11.8%)	2 (9.5%)	0.999

NA, not applicable; N, number; UL, unilateral; BL, bilateral; MRC, Medical Research Council’s; R, range.

**Table 3:** Secondary refinement procedures.

	UL Group	BL Group	*p*-Value
Donor sites, N (%)	17 (28.8%)	42 (71.2%)	NA
Aesthetic refinements donor site, N (% of donor thighs)			
Total	7 (41.1%)	10 (23.8%)	0.182
Scar correction	1(5.9%)	5 (11.9%)	0.662
Dog ear excision	0 (0%)	5 (11.9%)	0.308
Contour alignment donor thigh	2 (11.8%)	0 (0%)	0.079
Contralateral thigh alignment	4 (23.5%)	0 (0%)	0.005 *

NA, not applicable; N, number; UL, unilateral; BL, bilateral; * indicates significant difference (*p* < 0.05).

**Table 4:** Aesthetic outcome.

	Unilateral Group	Bilateral Group	*p*-Value
Donor sites, N (%)	17 (28.8%)	42 (71.2%)	NA
Scar length (cm), Mean (SD)	21.3 (3.8)	22.9 (3.1)	0.891
Scar position to the groin (cm), Median (R)	1.0 (0.2–3.5)	1.0 (0.3–4.5)	0.329
Thigh circumference (cm), Mean (SD)			
Proximal thigh			
Donor leg vs. contralateral leg			
Donor leg vs. donor leg	59.5 ± 3.9 vs. 61.9 ± 4.6		0.118
Mid-thigh		59.3 ± 6.3 vs. 60.5 ± 6.4	0.558
Donor leg vs. contralateral leg			
Donor leg vs. donor leg	55.6 ± 4.6 vs. 56.4 ± 4.6		0.597
Lower thigh		55.3 ± 4.8 vs. 56.3 ± 4.9	0.481
Donor leg vs. contralateral leg			0.597
Donor leg vs. donor leg	49.5 ± 4.7 vs. 49.6 ± 4.9	48.4 ± 4.3 vs. 49.4 ± 4.4	0.453

NA, not applicable; SD, standard deviation; R, range; vs., versus; N, number.

**Table 5:** Patient-reported outcome measures.

	Unilateral Group	Bilateral Group	*p*-Value
Patients, N (%)	17 (44.7%)	21 (55.3%)	NA
LEFS (points), Median (R)	s	78.5 (47–80)	0.120
BODY-Q Inner Thigh * (points), Median (R)	66 (33–100)	66 (0–100)	0.487
BODY-Q Scar ** (points), Median (R)	65 (18–100)	66 (0–100)	0.791
BODY-Q Sexuality *** (points), Median (R)	47 (0–86)	49 (0–75)	0.902
Pain, N (%)			
at rest	0 (0%)	3 (14.2%)	0.238
during movement	6 (35.3%)	8 (38.1%)	0.858
Labial spreading, N (%)	2 (11.8%)	1 (4.8%)	0.576

NA, not applicable; N, number; R, range; *, BODY-Q Satisfaction with the inner thighs; **, BODY-Q Appraisal of scars; ***, BODY-Q Sexual well-being; LEFS, Lower Extremity Functional Scale.

## Data Availability

The data presented in this study are available on request from the corresponding author. The data are not publicly available due to ethical, legal and privacy issues.
